# Cognitive behavioral therapy for psychosomatic problems in dental settings

**DOI:** 10.1186/s13030-017-0102-z

**Published:** 2017-06-13

**Authors:** Hirofumi Matsuoka, Itsuo Chiba, Yuji Sakano, Akira Toyofuku, Yoshihiro Abiko

**Affiliations:** 1 Division of Disease Control and Molecular Epidemiology, Department of Oral Growth and Development, School of Dentistry, Health Sciences University of Hokkaido, Ishikari-Tobetsu, Hokkaido Japan; 2 School of Psychological Science, Health Sciences University of Hokkaido, Ishikari-Tobetsu, Hokkaido Japan; 3 Department of Psychosomatic Dentistry, Graduate School of Medical and Dental Sciences, Tokyo Medical and Dental University, Bunkyo-ku, Tokyo Japan; 4 Division of Oral Medicine and Pathology, Department of Human Biology and Pathophysiology, School of Dentistry, Health Sciences University of Hokkaido, Ishikari-Tobetsu, Hokkaido Japan

**Keywords:** Cognitive behavioral therapy, Temporomandibular disorder, Dental anxiety, Burning mouth syndrome, Atypical odontalgia, Halitophobia, Dry mouth

## Abstract

Cognitive behavioral therapy (CBT) has been applied for various problems, including psychiatric diseases such as depression and anxiety, and for physical symptoms such as pain. It has also been applied for dental problems. Although the effect of CBTs on temporomandibular disorders and dental anxiety are well documented, its effectiveness on other types of oral symptoms remain unclear. Little information comparing the different types of CBTs in the dental setting is currently available. Because dental professionals are often expected to conduct CBTs in the dental setting, it is important to develop proper training programs for dental professionals.

In this review article, we demonstrate and discuss the application of CBTs for psychosomatic problems, including temporomandibular disorders, dental anxiety, burning mouth syndrome, and other oral complaints in dental settings.

## Background

Cognitive behavioral therapy (CBT) is a psychological intervention that has been applied for various health-related issues, including psychiatric diseases such as depression and anxiety, and for physical symptoms, such as pain disorders [1, 2]. The number of studies that have examined the effect of CBT on these problems are increasing [1]. CBT has been proven more effective than other means of psychotherapy for the treatment of psychiatric disorders [3]. Moreover, systematic reviews of CBT have demonstrated an increase in the number of meta-analyses from 10 in the early 2000s to approximately 40–50 within the following 10 years.

Recently, CBT has begun to be applied for psychosomatic problems in the dental setting, and the effectiveness of this therapy on these problems has been confirmed in various studies [4–10]. Herein, we review these previously conducted studies and discuss the effectiveness of this therapy for adult patients with psychosomatic dental problems.

### Cognitive behavioral techniques used in dental settings

The basic premise of CBT is that physical or emotional problems are difficult to change directly, so CBT targets these problems by changing cognitions and behaviors that are contributing to the physical or emotional problems. Changing cognition and behavior can be made using the following techniques.

- biofeedback
  Biofeedback is a form of treatment to improve physiological functioning by the use of monitoring the equipment that provides the patient with real-time information regarding specific symptom-related biological response. Biofeedback, particularly electromyography biofeedback, is frequently used for temporomandibular disorder. In this treatment, patients practice keeping their muscle (e.g. masseter or temporal muscles) relaxed though monitoring their muscle activity.

b) relaxation
  Relaxation is a techniques to improve the various symptoms by relaxing the body, including progressive muscle relaxation, autogenic training, and breathing.

c) exposure
  Exposure is a technique to improve fear reactions by exposing patients in fearful objects or situations and not allowing them to use avoidance behaviors that might help reduce fear in the short term, but that would make fear worse in the long term. In dental settings, exposure is frequently used for patients with dental anxiety. These patients fear situation or stimulus related to dental treatment, including sitting in the treatment chair, opening the mouth, the use of a mirror in the clinical exam, injection of local anesthesia, and the drilling of a cavity.

d) cognitive restructuring
  Cognitive restructuring is a technique to identify and modify maladaptive thoughts related to emotional and behavioral problems. One of the cognitive factors targeted in dental settings is pain catastrophizing. These factors are treated using an automatic thought record. Using this tool, patients can identify, evaluate, and modify their thoughts.

### Temporomandibular disorder (TMD)

Temporomandibular disorder is a heterogeneous collection comprising pain and dysfunction in the muscles used for mastication or in the temporomandibular joints [11]. The prevalence of TMD in a community sample was almost 17.5%, although various values have been reported previously [12, 13]. In a recent review article, biofeedback was reported to be more effective than active control or no treatment in reducing TMD symptoms [4]. CBT, including cognitive intervention, was more effective than conventional CBT or no treatment [4]. The effectiveness of biofeedback in the short term and that of CBT in the long term have been confirmed by meta-analyses (Table 1). Although CBTs were mainly conducted by psychologists (Table 2), those conducted by dental hygienists who had received 8 h of CBT training were also found to be effective in reducing TMD pain and pain-related interference [14].

**Table 1:** Results of meta-analyses of dental complaints

Authors	Year	Number of studies	Treatment content	Comparison for meta-analysis	Effect size^a^			
					Short term		Long term	
TMD					pain	clinical exam^b^	pain	clinical exam
Crider and Glaros [6]	1999	13	EMG biofeedback	control (psychological placebo or no treatment)	0.47	0.26	-	-
Roldan-Barraza et al.[5]	2014	12	cognitive behavioral therapy	control (usual treatment)	0.07	-	0.66	-
Dental Anxiety					self-reported anxiety	dental attendance	self-reported anxiety	dental attendance
Kvale et al.[8]	2004	38	cognitive behavioral therapy	control (anesthesia/sedation or no treatment)	1.78	1.4	-	1.17
Wide Boman et al.[9]	2013	10	cognitive behavioral therapy	control (anesthesia/sedation)	2.02	-	2.25	-
				control (no treatment)	3.26	-	-	-

^a^The effect sizes were reported in each articles and were calculated by subtracting the mean of the control group from the mean of the treatment group at post-treatment and dividing by the pooled standard deviation of the two groups. An effect size of 0.2 represents a small effect, 0.5 a moderate effect, and 0.8 a large effect (Cohen, 1988) [48]

^b^Clinical exam is the measures derived from an examination of the temporomandibular joint and masticatory muscles. This category could include single measures of muscle palpation pain as well as combined measures of palpation pain and additional observations such as temporomandibular joint function, temporomandibular joint pain, and mandibular mobility

**Table 2:** Practitioner and training methods in treatment studies

Authors	Year	Number of participants	Practitioner of CBT	Training for practitioner	Number of sessions	Treatment contents
TMD (Roldan-Barraza et al., 2014) [5]						
Dworkin et al. [49]	1994	139	psychologist/dentist	trained in the use of materials and methods and with pilot treatment session	2 sessions	psychoeducation, self-monitoring, stress-coping, PMR
Dworkin et al. [50]	2002	117	clinical psychologist experienced in the use of CBT methods with chronic pain	followed a written manual for each session	6 sessions	psychoeducation, cognitive restructuring, relaxation, coping
Dworkin et al. [14]	2002	124	hygienist	8 h training	3 sessions	psychoeducation, relaxation, stress management, self-monitoring
Ferrando et al. [51]	2012	59	psychologist with 2 years of clinical training	protocol was standardized	6 sessions	psychoeducation, distraction, hypnosis, cognitive restructuring, assertiveness training
Litt et al. [52]	2010	101	master’ level therapists with at least 2 years of experience in CBT	protocol was standardized and all sessions were audiotaped and evaluated.	6 sessions	relaxation, stress management, cognitive restructuring
Turner et al. [53]	2006	158	licensed clinical psychologists	trained and supervised in the protocol	4 sessions	cognitive restructuring, PMR, abdominal breathing, fear-avoidance discussion
Dental Anxiety (Wide Boman et al., 2013) [9]						
Berggren & Linde [54]	1984	99	-	-	5 sessions	systematic desensitization with biofeedback
Moses & Hollandsworth [55]	1985	24	predoctoral intern in clinical psychology	training in stress inoculation from senior author	1 sessions	stress inoculation training
Gatchel [27]	1986	40	videotaped program	-	1 sessions	systematic desensitization and coping skills training
Getka & Glass [56]	1992	41	graduate students in health psychology	10 h training with detailed manuals	6 sessions	modeling and systematic desensitization with audiotaped relaxation Stress inoculation training
de Jongh et al.[57]	1995	29	psychologist/dentist	-	1 sessions	cognitive restructuring
Willumsen et al.[22]	2001	62	dentist	5 patients were treated and video recordings of sessions were used for supervision	10 sessions	cognitive restructuring and exposure Applied relaxation
Haukebø et al.[21]	2008	40	dentist specially trained in CBT for dental anxiety	-	1 session vs 5 sessions	exposure and behavioral experiments

Table 2 also shows the kind of treatment in which CBT was used. A review on psychosocial treatments for TMD demonstrated that CBT is often conducted using multiple techniques, such as education, stress management, cognitive restructuring, and relaxation (Table 2). Although treatments that include multiple techniques might provide unnecessary techniques and increase the cost of treatment, the use of a simple CBT technique has not yet been reported [5]. Questions pertaining to the types of patients affected by multiple CBT techniques were evaluated in a qualitative systematic review [15], wherein CBTs were applied to three different groups of TMD patients; those presenting with disc displacement and no reduction in pain or major psychological symptoms (Group 1), those with TMD pain and no major psychological symptoms (Group 2), and those presenting with TMD pain along with major psychological symptoms (Group 3). In groups 1 and 2, the effectiveness of a single therapeutic approach was found to be similar to that of multiple combination approaches. On the other hand, the patients in Group 3 were found to be significantly affected by the combinations of therapeutic approaches. Turk et al. [16] examined the effect of a combination of stress management programs and biofeedback with CBT on Group 3 type patients and reported significant improvement in symptoms after treatment. These results imply that it is possible to decrease the treatment cost by providing a combined therapeutic approach for TMD patients with psychological problems and single therapeutic approach for patients with no psychological problems. Diagnosing the TMD of patients with psychological problems could be done using the Research Diagnostic Criteria for Temporomandibular Disorders (RDC/TMD) and introducing clinical (axis I) and psychological assessments (axis II) to the classification system [17, 18].

### Dental anxiety

Approximately 10–12% of the adult population suffer from dental anxiety [19, 20]. The effectiveness of CBTs with various relaxation, distraction, cognitive restructuring, systematic desensitization, and exposure techniques for dental anxiety have been confirmed [7–9]. In the 10 RCTs on CBT for dental anxiety reviewed by Wide Boman [9], a significant reduction in subjective anxiety was achieved by patients with CBT when compared to those who received no treatment or anesthesia/sedation. Moreover, the effectiveness of the therapy was maintained for one to two years [9]. In a previously reviewed paper about patients with dental anxiety, the majority of studies (33/35 articles) demonstrated that CBT reduced subjective anxiety [8]. As shown in Table 1, the effect sizes calculated based on these subjective anxieties were large (1.78–3.26 at post-treatment and 2.25 at follow-up (6 months to 1 year after treatment)).

In addition to subjective anxiety, the ability to visit a dental office by a person who previously could not was used as another indicator of dental anxiety; approximately 80% of patients who received CBT could visit the dental office within 6 months [8, 9]. The efficacies of CBT treatments were maintained at four years after the treatments, and 48–100% of patients could visit the dentist during that period [8]. The effect sizes calculated based on these dental attendances at post-treatment (1.4) and follow-up (1.17; 6 months to 4 years after treatment) were large (Table 1).

Again focusing on Table 2, in a review by Wide Boman [9], CBT programs were conducted by dentists in two out of seven RCT studies and by a clinical psychologist or graduate student specialized in clinical psychology in the remaining four studies. Of the two studies in which CBT was conducted by a dentist, one reported that the dentists had five years of experience in treating dental anxiety patients using this method [21], whereas in the other study, the dentists, supervised by a clinical psychologist who used a video recording of the sessions, received training sessions in which they were required to conduct CBT, based on detailed manuals, for five patients [22]. Considering the effectiveness of CBTs conducted by dentists [9], the reason for the small number of dentists using it is the lack of training. Hence, it is advisable that dentists receive further training in how to conduct CBT.

Also as shown in Table 2, various techniques are used in combination while treating dental anxiety patients by CBT. Those using the exposure technique are more effective when compared with treatments that do not use this technique [7]. Exposure was consistently effective despite variations, such as individual vs group or image vs in vivo; therefore, it did not matter which different techniques (relaxation or cognitive component) were paired with the exposure technique [7]. Moreover, the exposure technique was found to be equally effective for dental anxiety as for other types of specific phobias [23].

A factor that can be used to determine the treatment procedures for dental anxiety is the severity of the symptoms [24]. To the best of our knowledge, there are no systematic reviews on whether or not the intensity of anxiety has any effect on the efficacy of CBT. The average score of Corah’s Dental Anxiety Scale (DAS) [25] exceeded 15 points in almost all studies in a recently reviewed paper [9]. Because patients with severe dental anxiety scored more than 15 points on the DAS [26], almost all subjects in the studies cited in the review were patients with severe anxiety. The effects of CBT were compared between patients with high DAS scores (16.6) and those with moderate scores (11.5) [27]. Improvement following a dental visit was more prominent in patients with moderate anxiety than in those with severe anxiety, although no difference in reduction of anxiety was observed between the two groups [27]. Thus, CBT may more effective in improving the behavior outcomes, such as visiting a dental office, of patients with moderate than with severe anxiety.

### Burning mouth syndrome (BMS)

Burning mouth syndrome is characterized by a burning sensation or other dysesthesias of the oral mucosa, unaccompanied by any other abnormal clinical or laboratory findings. BMS, with a prevalence of 3.7 ~ 7.9% [28, 29], is also known as stomatodynia, stomatopyrosis, glossodynia or oral dysesthesia. The definition offered by the International Headache Society presumes that BMS is idiopathic by nature; attempts are being made to identify risk factors connected with the syndrome’s etiopathogenesis [30]. Because more than 50% of the patients with BMS sustain their symptoms for prolonged periods even after several treatment methods [31, 32], various other methods including psychological and pharmacological approaches have been applied for BMS [10].

CBT is recommended as a therapeutic approach for BMS [10]. Twelve to 16 sessions of CBT improved the pain severity and discomfort of patients with BMS, and the effects were maintained 6 to 12 months after therapy [33, 34]. CBT conducted as a form of group treatment with short durations (1–2 sessions) also improved the pain and anxiety of the patients; these treatments focused on reducing dysfunctional cognitive factors [33, 34]. In a recent study, we demonstrated that a cognitive factor such as pain catastrophizing influences pain severity and oral health-related QOL [35]. CBTs with interventions that focus on the alteration of pain-related catastrophizing (Table 3) dramatically improved BMS symptoms (56%, very much improved; 44%, minimally improved) [36]. A previous study showed that the condition of 40% of the patients with BMS was improved by other types of CBTs without focusing on the alteration of pain-related catastrophizing [34]. Therefore, CBTs that focus on pain-related catastrophizing might be more effective for BMS patients when compared to those that do not.

**Table 3:** Contents of treatment focusing on the alteration of pain-related catastrophizing

	Treatment contents
Session 1	Psychoeducation for burning mouth syndrome
	Provide information on symptoms of burning mouth syndrome, possibility of serious disease, averse effect of cognition and feeling on pain, strategies used in treatment program.
	Progressive muscle relaxation
	Self monitoring of pain symptoms
Session 2	Distraction
	Identification of automatic thoughts in painful situation
	Practice monitoring feeling and automatic thoughts (catastrophizing thoughts).
Session 3	Evaluation automatic thoughts
	Practice evaluating of automatic thoughts
Session 4	Replacement of automatic thoughts
	Practice replacing catastrophizing thoughts with more adaptive thoughts

### Other oral complaints

CBTs are thought to be effective for the treatment of other oral symptoms, such as atypical odontalgia (AO), which is a subgroup of persistent idiopathic facial pain disorder as defined by the International Headache Society [30]. Patients with AO complain of medically unexplained toothache. As with other chronic pains, the effectiveness of anti-depressants including nortriptyline and milnacipran have been confirmed for AO [37, 38]. Although the effectiveness of CBT on some types of orofacial pains have been reported earlier [39], this therapy has not as yet been applied to AO. We believe that CBT is useful for the treatment of patients with AO; however, further investigations exploring the effectiveness of this treatment on AO are required.

Halitophobia is associated with anxiety in the dental setting and is categorized as Olfactory References Syndrome (ORS). ORS is a persistent false belief of the presence of body odor resulting in significant distress and functional impairment [40, 41]. The basic characteristics of ORS are similar to those of body dysmorphic disorder and social anxiety disorder [40]. Because CBTs have been proven effective for both body dysmorphic and social anxiety disorders [1], they may be effective for the treatment of halitophobia.

The symptom of oral dryness, called dry mouth, is related to psychological factors [42, 43]. Salivary flow rates and other characteristics are controlled by autonomic nerves. Stress decreases salivary flow and causes the formation of viscous saliva via predominance of sympathetic nerves [44]. These conditions often lead to complaints of oral dryness, especially during anxiety and depression [42, 43]. Therefore, the roles of cognitive appraisal and coping for stressors have been explored in patients with dry mouth [45]. Patients with Sjögren’s syndrome used maladaptive coping more frequently and have less social support when compared to those with lymphoma and healthy controls [45]. We have previously shown that the cognitive style of patients with dry mouth is correlated with their oral health-related QOL; 19% of patients presented with negative cognitive styles [46]. The results of that study indicated that interventions designed to alter the cognitive style of dry mouth patients might improve their oral health-related QOL. Because approximately 50% of the patients who complained of oral dryness demonstrated a salivary flow rate lower than the diagnostic standard of Sjögren’s syndrome [47], we believe that psychological factors may greatly influence the symptoms of patients with dry mouth. Psychological approaches, including CBT, may be useful in the treatment of dry mouth. Thus, further investigations are needed to develop effective psychotherapy measures for these patients.

## Conclusion

In many intervention studies, the effectiveness of CBT for psychosomatic problems in dental settings have been confirmed. Although the effectiveness of this method for TMD and dental anxiety are well documented, its effectiveness on other types of oral complaints are poorly explored. In addition, there is a lack of information regarding the suitability of the various types of CBTs for different kinds of patients. The small number of reports on CBT in the dental setting may be due to the unavailability of CBT specialists. Dental professionals need to be skilled enough to be to conduct CBTs as efficiently as psychologists; therefore, it will be important in the future to develop proper CBT training programs for dental professionals.

## Abbreviations

AO: Atypical odontalgia
BMS: Burning mouth syndrome
CBT: Cognitive behavioral therapy
DAS: Dental anxiety scale
ORS: Olfactory References Syndrome
TMD: Temporomandibular disorder
